# Effects of incarceration on risky Sex: focus group data from Two New England states

**DOI:** 10.1186/2194-7899-2-8

**Published:** 2014-04-02

**Authors:** Marlanea E Peabody, Adam Choung, Rochelle Rosen, Caroline Kuo, Wendee Wechsberg, Karen Fernandes, Caron Zlotnick, Jennifer Johnson

**Affiliations:** 1 Brown University, Box G-BH, Providence, RI 02912 USA; 2 The Miriam Hospital, Centers for Behavioral and Preventive Medicine, Coro West, Suite 309164 Summit Ave, Providence, RI 02906 USA; 3 121 S. Main St., 4th Floor, Rm 406, Providence, RI 02903 USA; 4 RTI International, PO Box 12194, RTP, NC 27709-2194 USA; 5 345 Blackstone Blvd.,, Providence, RI 02906 USA

**Keywords:** HIV, Incarceration, Female, Prisoners, Safe sex, Women, Justice

## Abstract

**Background:**

Human Immunodeficiency Virus (HIV) risk and interpersonal violence are interconnected public health problems facing incarcerated women. Prison may provide an opportune time to conduct HIV prevention activities with high-risk women.

**Methods:**

This study used qualitative analysis to explore how incarceration affected women’s experiences of and thoughts about sex and sex risk. Twenty-one incarcerated women who had engaged in unprotected sex with a male in the 90 days prior to incarceration and experienced interpersonal violence in their lifetime participated in semi-structured focus groups at four women’s prison facilities in two New England States.

**Results:**

Themes that emerged from these focus groups include: a) incarceration increased sexual desire for some women but decreased it for others, b) education and exposure to women with HIV during incarceration increased women’s intentions to use condoms after release, c) women recognized that partners were often unfaithful while women were incarcerated, d) women felt empowered by mental health/substance use treatment and sobriety in prison, and e) practical difficulties of re-entry challenged women’s resolve to practice safe sex after release.

**Conclusion:**

Themes illuminate possible directions for public health interventions for this population at high risk for HIV.

**Electronic supplementary material:**

The online version of this article (doi:10.1186/2194-7899-2-8) contains supplementary material, which is available to authorized users.

## Background

Recent data suggests that there are over 200,000 women in U.S state and federal prisons and jails on any given day (Golinelli and Carson 2013). Moreover, there are an additional one million women on probation or parole (American Civil Liberties Union 2013). Women represent one of the fastest growing populations in federal and state prisons (Golinelli and Carson 2013; American Civil Liberties Union 2013 Henderson 1998). Convictions for drug crimes and other drug related offenses have greatly contributed to this rise in incarceration rates (Henderson 1998).

From an epidemiological standpoint, incarcerated women represent a unique population with distinctly higher prevalence of HIV and other sexually transmitted infections (STIs) compared to male inmates and women in the general community (McQuillan and Kruszon-Moran 2008; Henderson 1998; Datta et al. 2007; Gottlieb et al. 2008; Clarke et al. 2006; Hale et al. 2009; United States Department of Justice 2005). Studies have indicated that women offenders are 15 times more likely to be infected with HIV than women in the general community, and are more likely to be infected with HIV and STIs than incarcerated men, making incarcerated women an important target population for HIV prevention initiatives (McQuillan and Kruszon-Moran 2008; Henderson 1998; Datta et al. 2007; Gottlieb et al. 2008; Clarke et al. 2006; Hale et al. 2009; United States Department of Justice 2005). The elevated HIV risk among incarcerated women has been attributed to various factors, including a history of past trauma, STIs, alcohol and drug use and abuse, sex with multiple partners, trading sex for money or drugs, mental health disorders, socio-economic conditions, and poor condom negotiation skills (Arriola et al. 2005; Blumberg and Dickey 2003; Breslau et al. 1997; Campbell et al. 2008; Davila and Brackley 1999; El-Bassel et al. 2001; Kalichman et al. 1998; Khantzian 1987; Klein et al. 2008; Koenig and Clark 2004; Maman et al. 2000; Mandell et al. 1999; Rodgers et al. 2004; Sareen et al. 2009). For example, more than half of incarcerated women in state correctional facilities report a history of prior sexual or physical abuse (Browne et al. 1999; Harlow 1999), a factor that is closely tied to HIV and STI risk in other populations of women (Arriola et al. 2005; Breslau et al. 1997; Koenig and Clark 2004; Rodgers et al. 2004).

Few studies, however explore the effects of incarceration on risky sexual behavior and HIV risk. Three studies that focused on male incarceration found that incarceration was related to the dissolution of the primary sexual and emotional partnership (Epperson et al. 2011; Khan et al. 2011a 2011b). Further, this dissolution was correlated with released men having an increased number of sexual partners, increased number of unprotected sexual occasions after release, and increased incidence of other STIs (Khan et al. 2011a 2011b). More specifically, dissolution of the primary partnership is associated with a 3 times greater risk of having 2 or more sexual partners after release, after adjusting for socio-economic status and crack cocaine use (Khan et al. 2011a). One study on methadone-using male prisoners noted the importance of the bi-directional risk between male prisoners and their female partners, who may also be involved in the criminal justice system (Epperson et al. 2011). Results indicated that male prisoners with incarcerated female partners were likely to have multiple partners in the 12 months post incarceration (Epperson et al. 2011). Overall, there are few existing research studies that study how incarceration might impact sexual risk behaviors, and all of these studies have focused exclusively on incarcerated men. While these studies suggest that the experience of incarceration have effects on subsequent sexual behavior among men, effects may be different for incarcerated women, particularly due to unique gendered experiences and decisions about sex.

The purpose of this exploratory qualitative study is to explore women’s perspectives of how incarceration has affected their thoughts about and experiences of sex and sex risk. Given this population’s high prevalence of HIV infection and HIV/STI risk factors (i.e., sexual and physical abuse), it is important to understand their unique experiences in order to incorporate these factors into the design of future interventions.

## Methods

### Participants

Four focus groups (90 minutes each) were conducted, one at each of 4 different women’s correctional facilities in 2 New England states. These 4 facilities (one minimum security and one medium security facility in each state) contained the entire female state prison populations in both states. Participants were English-speaking female volunteers. The target population was incarcerated women over the age of 18 who: a) had at least one unprotected sexual encounter with a male within 90 days prior to incarceration (as assessed by the Timeline Followback for Sex Risk Behaviors, which uses a calendar-based interviewing method to increase recall by recording protected and unprotected sexual behavior by partner each day during the target period, with excellent reliability and validity; Sobell and Sobell 1995; Stein et al. 2001 2002), b) reported a history of lifetime physical or sexual abuse as assessed by a “yes” response to any of the Physical or Sexual abuse subscale items of the Trauma History Questionnaire interview (Green 1996), and c) were willing and able to participate in a focus group during the scheduled day and time. These criteria identified participants that were at an increased risk of contracting HIV and other STIs by virtue of a history of unprotected sex and interpersonal violence. Focus groups were designed to be formative research to develop an intervention for incarcerated women with a history of interpersonal violence victimization (i.e., sexual or physical abuse or assault), a group at particularly high risk for HIV. This formative research enrolled participants separately from the subsequent intervention study; therefore, participation in the current study consisted only of answering baseline questionnaires and participation in one 90-minute focus group.

Participants who met inclusion criteria (n = 25) and who attended the focus group session (n = 21) were included in analyses. Their mean age was 34.85 years. Participants were non-Hispanic white (80%), Hispanic (10%) or mixed race (10%), which is roughly representative of incarcerated women in New England. The majority of the participants were serving short-term prison sentences, although sentences ranged from 90 days to 9 years in prison. Participants had been incarcerated for an average of 66.36 weeks and that value ranged from 1 week to 872 weeks. Offenses for current incarceration ranged from prostitution (90 days) to embezzlement (9 years). These sentences reflect the amount of time ordered to be served in prison, and not additional probation time.

### Procedures

Participants were recruited via an announcement made at each prison facility. Trained research staff recruited participants via announcements in the housing units and other common prison areas of each prison facility. Announcements described the study’s need for volunteers who would be willing to talk about women’s experiences of health and incarceration. Potential participants were instructed to complete a confidential slip and then submit the slip to the research staff. Those who indicated interest were then called down individually to meet privately with a research team member. In this meeting, women were screened in order to determine eligibility. A trained research staff member read aloud the consent form; participants were then asked to sign a copy of the consent form. Each participant was provided with a copy of the consent form. Research protocols were approved by the Brown University Institutional Review Board and each state’s Department of Corrections review committees.

Eligible women were then invited to participate in the focus groups. The focus groups were semi-structured and lead by a moderator; a second member of the research team took notes. Focus group moderators encouraged open discussion by: a) explaining that the purpose of the research was to get information about the thoughts and experiences of incarcerated women in order to design a new intervention to women’s HIV and revictimization risk and that participants’ honest answers would help us do that; b) emphasizing that there were no right or wrong answers; c) emphasizing that researchers valued each woman’s ideas and wanted to hear from all participants; d) explaining confidentiality carefully; e) asking less threatening questions first; and f) asking about how “incarcerated women” (in general) think about sexual health, sexual behavior, HIV/STI risk, substance use and violence (see Table 1), not necessarily any single woman in particular. Our experience with incarcerated women in previous research (Johnson et al. (2013); Johnson et al. (in press)) is that many are open to sharing their opinions and their stories, especially if they feel they will be heard and respected, and that doing so might help another woman in a similar situation. The groups were audio recorded and these recordings were transcribed using a secure transcription service. There were no legal or financial incentives for participation.

**Table 1:** **Structure of moderated focus groups**

Topic	Estimated time	Question examples
Introduction	5 minutes	• Basic introduction
HIV risk	25 minutes	• How do women choose sexual partners when they leave prison?
		• What’s the goal for sex?
		• Do women see the same men from before coming to prison?
		• Do you think that they are good partners and what makes them so?
		• Are they not so good? What makes them bad partners?
		• Do you think women leaving prison use protection?
		• Do you think women can talk to their men about whether other women are in the mix?
		• Do you believe that woman have problems protecting themselves?
		• What about male partners who are using drugs or drinking, is it hard for women to use protection then?
		• Do you believe that women have problems protecting themselves when they use drugs?
		• How do women get HIV?
		• How can women protect themselves from HIV?
Condoms	20 minutes	• Why don’t women use condoms more?
		• Can you make (condoms) sexy?
		• Do you think women are afraid of partners being violent when women bring up condom use?
Sexual situations, safe sex and violence	30 minutes	• When women want to use protection but don’t, what gets in their way?
		• When women are about to have sex, what emotions do they feel?
		• Does have a supportive person in your life change how confident you feel asking a partner to use protection?
		• What happens when women have had violence in their lives?
		• Who controls sexual situations?
		• themselves? How do women stay strong?
		• What can women do to stay strong and empower
The end	10 minutes	• Summary

### Data analysis

The transcribed and de-identified versions of the focus group recordings were imported into an NVivo database. One graduate level public health student and one bachelor level research assistant first analyzed each transcript and identified important themes in the data relating to ways in which the experience of incarceration affected women’s thoughts and perceptions of sex and sex risk. Using these individually identified themes, they created an initial codebook. Each individual then coded each transcript again with this codebook. The codebook was continuously revised in order to better meet the needs of the study. Each rater coded the 4 transcripts, and then both met to compare codes and to revise the codebook. All discriminant codes were discussed and a consensus was reached in order for the final code to be recorded. This process was completed 3 separate times. The final codebook consisted of 18 root codes and 7 sub additional codes (See Table 2). The final codebook was then imported into NVIVO, and all transcripts were electronically coded with this software. Analysis focused on how experiences of incarceration related to HIV risk behaviors, substance use, empowerment, and re-entry into the community. Although 5 themes were selected for the results section, it is important to note that themes are not necessarily discrete and there is some overlap between themes.

**Table 2:** **Themes and sub themes**

Number	Theme name
1	Changing attitudes about sex because of incarceration
1a	Less interested in sex
1b	More interested in sex
2	Concerns about partner’s faithfulness while one is incarcerated
3	Sex for survival and sex as a coping mechanism
4	Reported mental health issues
5	General thoughts on condom use and safe sex
5a	Idea of wanting to become infected
5b	Being afraid and fearful of HIV and STIs
6	HIV/STI Testing
7	Strategies to getting a male partner to use a condom after incarceration
8	Reasons why women use condoms
9	Reasons why women do not use condoms
10	The relationship between drug use and sexual behavior, with condom use
11	Number of sex partners, before and after release
11a	Monogamous relationships
11b	Multiple sexual partners
12	Relationship with children
12a	Children has a motivator to stay clean and to improve life after prison
12b	Desire to teach children about safe sex
13	Communication with sexual partners about sex
14	Empowerment
15	Past family environment, abuse, and domestic violence
16	Access to community resources post release
17	Interpersonal violence and abuse
18	Coping skills

## Results

Five main themes related to the effects of incarceration on women’s experiences and thoughts of sex and sex risk emerged from the analysis: a) incarceration increased sexual desire for some women but decreased it for others, b) education and exposure to other women with HIV during incarceration increased intentions to use condoms after release, c) women recognized that partners were often unfaithful while women were incarcerated, d) women felt empowered by mental health/substance use treatment and sobriety in prison, and e) practical difficulties of re-entry challenged women’s resolve to practice safe sex.

### Theme 1: effects of incarceration on sexual desires

For many, incarceration forced abstinence from sex and sex within the primary partnership. This abstinence translated into an a) increased need and desire for sex after release for some women, but also b) decreased desire for sex for others. Both have implications for safe sex and condom use.

#### Increased desire for sex after release

Some women spoke of a strong desire to have sex after release because of forced abstinence during incarceration. Some women noted that they would use their partners for sex after release because of this increased desire, as long as they perceived that their partners had remained HIV and STI free, *“Honestly, I’m pregnant, so my hormones are nuts, and I need to have sex, honestly, that’s all I want to do right now. I broke up with my boyfriend, but I’m probably gonna end up using him anyway, as long as he produces the correct paperwork [i.e., a clean HIV/STI test] because I don’t know what he’s doing out there now (ID 20)”.* Sex after release was sometimes planned and thought about, “*For myself, I've been incarcerated a year. I've been writing and planning for it right up until the day I get out (ID 28)”.* Other women noted the challenges arising from forced abstinence, *“How can I be in jail for three, six months, without my boyfriend, without have sex? I don't have privacy to play for myself, whatever. I gonna to die’. That was the worst point I could think about (ID 41)”.*

#### Decreased desire for sex after release

On the other hand, some women noted that forced abstinence during incarceration decreased sexual desire. This seemed to be prominent for women that had been in jail or prison for long periods of time or who had served multiple sentences, *“After the first time I was locked up, that's all I thought about, was like having sex at a jail. Then I went back to a program and all I did was kind of snuck around. This time, I'm here for a longer time this time. At first that's all you think about, but now it's not so much (ID 39)”.* Some women also noted that this forced abstinence was empowering, *“I feel like I have—as more and more time goes by that I'm resisting sexual temptation, which for me is a really hard thing, I feel like I'm gaining power within myself, the longer that I'm abstinent, so that maybe the next time it will mean something and I won't make those fucked up decisions that I was making (ID 40)”.* Moreover, another women noted that she was nervous about sex after release because of continuous forced abstinence, *“I think it's gonna be awkward, regardless. I haven't had sex in so long I don't even know if I remember how to do it (ID 29)”.*

### Theme 2: Education and exposure to women with HIV during incarceration increased women’s intentions to use condoms after release

Participants indicated that the fear of contracting HIV and STIs increased as a result of knowledge gained in prison. The knowledge that women gained in prison included: a) desire to learn about HIV/STIs while incarcerated, b) knowledge of their own status and exposure to other women with HIV/STIs, and c) increased ability to assess personal risk because of increased awareness and knowledge.

#### Women expressed a desire to learn about HIV/STIs while incarcerated

Many women expressed a desire to learn about HIV/AIDs while they were incarcerated and spoke of the importance of classes related to HIV/AIDs and sex, *“ I think what you need to do is the 90 days, when a woman’s getting released, it would be mandatory that they have to take a sex education- I know it sounds silly, but a sex-ed, health-ed class (ID 21)”.* Moreover, women spoke of the importance of the ability to learn in prison: *“Knowledge is power, and I would really like to know this stuff (ID 9)”.* Women seemed to value opportunities to learn more about sex, sexual safety, and HIV/STIs while incarcerated.

#### Women increased knowledge of their own HIV/STI status and exposure to other women with HIV/STIs during incarceration

Women discussed learning their current HIV/STI status after being tested in prison and being empowered by both knowing their result and the result being negative, *“… I’ve gotten tested here, and made sure that everything was okay, and thank god, my lucky stars, I’m okay…( ID 2)”.* Women also noted that they were exposed to HIV positive women in prison. One woman noted that incarceration was the first time that they had met someone that was HIV positive: “*…I never even met anyone that had HIV until I came to prison…(ID 39)”.* This also seemed to prompt a new awareness of the reality of disease.

#### Women reported being better able to assess personal risk because of increased awareness and knowledge

Education and knowledge gained in prison also seemed to prompt a new understanding of personal risk, *“I didn’t [use condoms] when I was abusing drugs a lot, and I got lucky. I don’t have HIV or Hep C or anything, and I got lucky so I made conscious decisions to use protection. So, when I leave, I will use protection. There were times in my past that I did and when I didn’t, and I got lucky. Now I know that, I will [use condoms] (ID 28)”.*

### Theme 3: Women recognized that partners were often unfaithful while women were incarcerated

Concerns over partner’s behavior while women are incarcerated were prominent. Women spoke of a) fear of contracting HIV and STIs from unfaithful partners, b) desire to ask partners about other potential relationships, and c) getting partners to use condoms after release because of an awareness of other potential partners.

#### Women were fearful of contracting HIV and STIs from unfaithful partners

Many women recognized that while they were incarcerated, their partners were not 100% faithful, and because of that, they were fearful of contracting diseases from these partners; “*…even women that have partners that they’ve been with for awhile, they could be worried about like if they were cheating or something”* and *“See, we’re in here, we’re faithful because we have no choice. They’re out there, they don’t have to be faithful. I’m not gonna catch any kind of STD [after I’m faithful and he’s not] (ID 7)”.* This fear promoted the desire to engage in safe sex after release.

#### Women expressed a desire to use condoms but were concerned about the ability to make this happen

Some women also noted that they planned to ask partners about other sexual relationships that occurred during their incarceration. *“ I think I would want to know, that's why I'd ask. Like for my safety, I wanna know what he's doing. I'm not gonna put my life in danger just because I'm scared to ask a question (ID 29)”.* Although some women said that they did not want to know and would rather just assume partners were unfaithful, other women saw asking these sensitive questions as vital for self-protection after release.

#### Women planned to get partners to use condoms after release because of an awareness of other potential partners

Women were aware that partners often engaged in sex with others while they were incarcerated and because of that, knew that they needed to engage in safe sex practices in order to protect themselves. However, many women viewed this as a challenge and spoke of indirect negotiation tactics to get their partners to use condoms with them after release, *“It's so difficult because it makes me feel like trust, you know. If I say to my boyfriend, "I want you to wear a condom now because I was locked and I don't know what you did outside," he's gonna say, "No, trust me." No, I don't, so it's gonna be a problem. I need to find another excuse (ID 41)”.*

### Theme 4: Women felt empowered by mental health/substance use treatment and sobriety in prison

Women spoke of feeling empowered to practice safer sex by: a) prison substance use and mental health treatment and psychoeducation, b) being separated from negative outside relationships and having peer support in prison, c) reflection on the relationship of past victimization to subsequent high risk sexual behavior, and d) increased motivation for safer sex practices and safer partners after release because of increased awareness.

#### Empowerment for safe sex through treatment and psychoeducation

Some participants indicated that treatment programs they attended while incarcerated were vectors for their own empowerment; *“I’m learning a lot about myself from the self-help classes I am taking in here…It makes me feel empowered. I’m gonna go out there, and you have to know yourself. I know it’s gonna be hard and I’m okay with that, but it’s gonna be better than being in here (ID 21)”.* Some women saw these prison treatment experiences as transformative: *“I am a whole different person than I was when I walked in these doors, I can tell you that (ID 1)”.* Women noted a need for treatment to help them change the way they saw and valued themselves: *“I think that you should like give a course on—women need to learn how to love themselves when they leave, because if you don’t love yourself, you’re not gonna respect yourself, and you’re not gonna do anything to protect yourself (ID 20)”.* Women also spoke of a greater awareness of the harm that engaging in risk behaviors can cause: “*I’ve noticed over the past few months, doing work on myself, that I value myself, but the risks I take- I can do more dope than this whole unit. I can drink you all under the Table. I’ll bet you I’ve slept with more guys than…That’s my armor, when in reality, those are my flaws. It’s like I’m kind of at a point of self-discovery, where I’m getting to know myself… (ID 40)”.* Women viewed this newfound self-understanding as a motivator and facilitator of safer sex in the future.

Temporary change in social networks led some women to feel more empowered to make safer sexual choices*.* The prison seemed to foster self-growth by forcing sobriety and separation from negative outside forces such as abusive partners, *“ninety-nine percent of the time when you come in a place like this, you end up alone. The people you thought were your friends or the ones you thought loved you the most, they’re nowhere to be found. No letters. No visits. No nothing. So, you’re alone (ID 24)”*. Women spoke of the ability to grow in prison with peer support of other inmates, *“with the programs and the strengths of other [incarcerated] women (ID 29)”.* Sobriety and commonality seemed to help unify many women, *“ the people in here know me better than my friends out there’ cause I was always high or drunk or whatever. It’s different (ID 24)”* and *“we came in together (ID 28)”.* Women noted that this was important, *“ I think other women are helpful to each other (ID 26)”.* This was integral to overall progress, empowerment, and self-esteem, *“You know what I mean because for so long, we allow people to hurt us and treat us wrong and whatever. Until we get some self-esteem and learn the things that make us tick and how we feel about ourselves, can we teach other people what we'll accept and how we'll allow them to treat us? We teach people how they can treat us. You know what I mean?(ID 28)”* Prison protected women from the realities of the outside world and fostered reflection: “ …*So, once you sit with self and you figure out what you’re willing to accept and what you need to change, then go like [another woman] said, go out, and find the help that you need and that’s what we both did (ID 24)”.* By attending classes in prison and seeking support of other inmates, women were able to see themselves as empowered survivors of their experiences, *“I’m a survivor of rape. I’m a survivor of domestic violence. I’m a survivor on the street (ID 18)”*. These recognitions changed some women’s perception of themselves and desires for their relationships, including safer sex.

#### Time to reflect on the relationship of past victimization to subsequent high-risk sexual behaviors

Prison presented an opportune time for women to reflect on past victimization history through treatment, psychoeducation, and peer contact, “*I think that the violence in my life, a lot of my childhood trauma and stuff, led out to me believing that in the end there is only death. It made me really reckless, so I didn't care about anything. It's all going to turn out the same. No matter which way I get there, I'm always going to get to the same place. The things that were violent that happened to me when I was a lot younger play a big part in what's going on now. I'm just figuring all of this out. They made me really careless to anything [including risky sex] and really accepting of really bad things (ID 40)*”. This history was often cited as a challenge relating to safe sex and partner choices. Women viewed this new awareness the effects of past victimization on current risky behavior as an important part of discussions about protecting themselves from HIV and STIs.

#### Increased motivation for safe sex practices after release because of increased awareness of factors leading to women’s sex risk

Many women spoke of prison as a time where they were able to reflect and understand the abuse that they had experienced on the outside through treatment, sobriety, and other support provided in prison, which sometimes motivated safe sex practices and limiting or ending relationships with abusive partners. This reflection often became a vector for empowerment, and many women were determined not to go back to abusive partners after release, “*Up until this jail term, I would never admit that. I'd be like, ‘Well, this happened to me when I was a kid.’ I'd make all kinds of excuses for my sexual misconduct, being a prostitute, being a drug addict. It was everybody else's fault and choice but mine. It's not. It's my choice. I'm really going to start making all the decisions when I leave jail. I don't think that getting a disease is going to be okay anymore (ID 40)*.” Changing attitudes related to being able to think about and understand past victimization history seemed to increase motivation for behavior change after release, including not going back to an abusive partner post release.

### Theme 5: Practical difficulties of re-entry challenged women’s resolve to practice safe sex

Women who had previously been incarcerated and released described numerous re-entry challenges that tested their resolve to avoid unsafe sex. In terms of safe sex resources, per se, women were generally aware of places to obtain condoms in the community such as at community clinics like Planned Parenthood, but some cited expense, or embarrassment and fear as barriers to access: *“Women don’t want to go to the store and buy them because they feel embarrassed…(ID 18)”*.

However, women were much more concerned about challenges to meeting other basic needs at reentry, some of which were the secondary consequences of incarceration. These challenges often shifted priorities away from safe sex. Women’s criminal histories created obstacles to accessing federal housing programs, qualifying for Medicaid or Medicare, applying for employment, or with obtaining student loans for post secondary education. For example, “*It’s like me right now, I got drug charges. Just because I got drug charges, I’m not allowed to do nothing out there, and I got kids. I don’t want to go back. I’m not- regardless, I’m gonna win my kids back, so I cannot go back to my old life. It’s gonna be so hard for me… I am not allowed to have Section Eight. I am not allowed to have housing. I’m not allowed to have a job because I got a felony…. So really, what’s out there for me? (ID 18)”*. Additionally, high need for community resources was often at odds with low availability: *“When you’re in a domestic violence situation, want to open their mouth, they want to call this 1-800 that they show on TV, and want to get help, but you got to signal, they say, or you got to repeat yourself three times. I don’t got the time of patience, or mentally, I don’t want to say is 20,000 times what I am going through. I need the help now and fast. There’s no really no one fast out there at all, at all, understand (ID 18)”.* Some women noted that they were released without clear linkage to post-prison care: “*they open the door and let you go, you’re gonna be right back living on the street… (ID 21)”.* Without clothes, housing, treatment, and legal employment, many women relapse to substance use/risky sex quickly and/or engaged in unprotected sex to meet their basic needs:

*“I have an IUD and that's known with the main male partners that I have. Me asking them to use a condom would be like saying, ‘What? Do you think I'm dirty? Is that what you think of me?’ I'd have to be like, ‘No, I think you're great’… Then that causes a rift, where I'm like, great, now I have to do damage control… Kind of like waiting on tables, like great, I just fucked up the order. Now I have to do table recovery, now I'm not going to get a tip. My tip is my stability, my money. When I say that men pay my bills, they pay my bills. I do not pay for a single thing. My phone, where I sleep, whatever I eat, everything is paid for by men… To me, getting a disease or not getting a disease that can or can't be curable is not worth me losing my house and my money (ID 40)”.*

## Discussion

Although women who are incarcerated are known to be at high risk for HIV and to have histories of risky sexual behavior, few previous studies have explored the effects of incarceration itself on women’s subsequent risky sexual behavior. Although most women expressed a strong preference to not be incarcerated, they acknowledged that incarceration may provide a window of opportunity in which high risk behavior is paused, they are safe from violent partners, and they can receive interventions that focus on condom negotiation, violence prevention, comprehensive sex education, and empowerment to female inmates. For others, prison offered classes, enabled them to gain peer support, and allowed them to access services that were otherwise unavailable to them. Prison created an environment to help women focus on themselves and work on previously unaddressed issues. Other studies have noted that prison presents a tremendous opportunity for intervention (Leukefeld et al. 2012; Mallory and Hesson-McInnis 2013; Scott et al. 2004; Staton-Tindall et al. 2007). Unexpectedly, women spoke of incarceration as having a positive effect of their subsequent risky sexual behavior. In fact, there are significant reductions in risky sex from before incarceration to after release among women in treatment as usual groups in randomized trials (Leukefeld et al. 2012; Mallory and Hesson-McInnis 2013; Staton-Tindall et al. 2007), providing quantitative evidence that incarceration may at least temporarily slow HIV risk behaviors during the first months after release.

However, participants also explained that the difficulties of community re-entry challenged their resolve and ability to follow through with safe sex in the community. In particular, our results suggest a great need for comprehensive post-prison aftercare and community services for this population, as the transition to the community presents multiple challenges (e.g., employment, training, education, family concerns including reunification with children, healthcare and substance use treatment access) which tended to take priority over safe sex. After discharge from prison, the numerous barriers to obtaining housing, education, employment, healthcare, and working with state child protective agencies create obstacles for recovery, safe sex, and overall health and safety (Buchanan 2006; Case et al. 2005; Sung and Richter 2006).

The finding that community resources available to ex-offenders are often insufficient for these individuals to maintain gains made in prison when they enter the community is consistent with findings in many other studies of health and health behaviors of re-entering individuals, especially women. Health behaviors plummet (Johnson (in press; Binswanger et al. 2012; Hammett et al. 2001; Mallik-Kane and Vishner 2008; Stephenson et al. 2005) and mortality spikes in the year after prison release (Binswanger et al. 2013; Zlodre and Fazel 2012). This is largely a policy failure. For example, Wang et al. (2013) found that food insecurity was associated with HIV risk behaviors among individuals re-entering the community from prison. Specifically, individuals who did not eat for an entire day were more likely to report risky sex (exchanging sex for money and sex while intoxicated or high) than were those who had at least a meal a day. Yet, individuals who have committed drug-related felonies are banned from receiving food stamps in many states. Similarly, research has concluded that addiction and problematic or abusive romantic relationships drive HIV risk behaviors among re-entering women (Staton-Tindall et al. 2007) and that post-release substance use and mental health treatment are needed for sustained recovery from these conditions (Butzin et al. 2005; Mallik-Kane and Vishner 2008; Martin et al. 1995). However, publicly funded treatment is either not present or difficult to access in many communities, especially for women without childcare or reliable transportation (Johnson et al. (in press; Kellett and Willging 2011; Ritchie 2001). Day-to-day survival (food, housing, a safe and legal way to make money) often takes precedence over treatment even for life-threatening medical conditions among re-entering populations (Johnson et al. (2013), Johnson et al. (in press; Stephenson et al. 2005; Blank 2013). Other research has noted that psychologically empowering women during incarceration does little in the long-term if women have little real power in the form of access to basic resources and safety in the communities to which they will return (Johnson et al. (in press; Kellett and Willging 2011; Ritchie 2001). It is well-established that strengthening health and other resources for justice-involved individuals in the community provides better long-term health outcomes and is more cost-effective than investing in incarceration alone (Wolff 2005; McVay et al. 2004; Natarajan et al. 2008). However, until communities can accommodate the health and practical needs of formerly incarcerated individuals, health gains made during incarceration (e.g., HIV risk, HIV treatment, mental health, addiction) will continue to be lost once former inmates return to the community (Draine et al. 2005).

Although the larger policy issues are arguably most important to address, our findings suggest several smaller steps that may also be helpful. First, incarcerated women in this study expressed an interested in obtaining education on sexual health, STIs, and STI prevention during incarceration. Providing this education during incarceration takes advantage of women’s interest, time, concerns about partner behavior, and the synergy with other treatment that they experience while incarcerated. In addition to treatment and education focused on relationships in general, STI and safe sex education could easily be offered. A small group setting might be best, where women who have been successful at locating condoms in the community or persuading partners to use them can share their experiences with other women. This education could emphasize how difficult re-entry can be and discuss strategies that are appropriate and realistic for sexual safety in the context of these challenges, such as avoiding sex while intoxicated or high to the extent possible and always carrying an interesting variety of condoms. In particular, interventions should take violence and victimization into account when designing safe sex interventions for incarcerated women. In our previous work, incarcerated women described a variety of indirect condom negotiation skills (e.g., strategies for slipping on a condom during oral sex without the man noticing, using flattery about the size of condom needed [regardless of the size of condom actually used], presenting female or other novel condom varieties as a way to “spice up” one’s sex life) as especially effective for increasing condom use without risking violence (Kuo et al. (under review)). Third, prisons could make women aware of a variety of locations to find male and female condoms in the community and provide this information in the list of resources found in many re-entry packets. Given that women said that some ways of accessing condoms post-release can be expensive and/or embarrassing, consider distributing safe sex kits (with sufficient variety of safe sex materials) to women at release and/or at locations in the community such as probation/parole offices, addiction and mental health treatment facilities, and other nontraditional locations. Fourth, public health efforts should also target high-risk men for condom use, with appropriate messaging for men (e.g., the “Real Men are Safe” program; Caslyn et al. 2009), including educating them about ways to make condom use “sexy” and fun, so that women releasing from prison do not face such an uphill battle to persuade their male partners to practice safe sex. Fifth, given the temporary shift in women’s social networks during incarceration, anything that can be done to influence the social networks they return to would be ideal. Couples therapy that discusses safe sex and addiction during partner visits to the prison presents logistical challenges but is possible in many facilities. Finally, comprehensive discharge planning services (including housing, employment, mental health, addiction treatment, paperwork such as identification and Medicaid coverage; Nelson, Perry, & Allen (2011)) should be offered within prisons and jails, but these programs also need to be integrated within the community to ensure a continuation of care for released women.

This study has both strengths and limitations. In terms of strengths, this study one of the only studies of our knowledge that explores the effects of incarceration itself on subsequent risky sexual behavior among incarcerated women. The focus groups provided a moderated, safe setting to confidentially discuss their views on HIV risk, condoms, sexual situations, safe sex and violence with the researchers and with each other. In terms of limitations, the majority of participants in this study identified as non-Hispanic Whites and relatively few identified as Mixed Race, no participants identified as African American. Though this is not uncommon in New England, these demographics are not representative of incarcerated women in some other areas in the country and are also not the most-at risk for HIV. Furthermore, volunteers for research studies may differ from other incarcerated women. Therefore, it is unclear the degree to which our results generalize to women incarcerated in other areas of the country, women of other races/ethnicities, and women who do not volunteer for research. Further research could address the effects of incarceration on HIV risk among women from different locations and demographics (race or ethnicity, rural/urban, prison vs. pretrial jail detention, etc) and examine ways to address the barriers to safe sex (especially the lack of community resources that can drive risky behavior as a means of survival) identified in this study. Two trials of a safe sex education intervention that was designed from this focus group data for re-entering incarcerated women who have experienced victimization are currently underway.

## Conclusions

Results provide some evidence that the experience of incarceration, the knowledge gained during incarceration, treatment during incarceration, and fears about what is happening outside prison while women are incarcerated, can positively shape incarcerated women’s thinking about HIV and STIs, their sex risk, and the importance of their own sexual safety. Findings suggest the following: a) prison presents an opportune time to provide HIV intervention classes to inmates because women are sober and away from abusive partners and other street dangers, b) increased access to treatment and other services in prison can impact safe sex after release by fostering empowerment and self growth, c) HIV testing in prison can provide women with the knowledge and education necessary to consider safe sex practices after release, d) concerns over partner’s status is prominent for incarcerated women and HIV intervention programs could incorporate this into an HIV risk reduction curriculum, e) there is a strong need for comprehensive counseling and support for incarcerated women offenders with histories of interpersonal violence, and f) there is an even greater need for more comprehensive post release aftercare as well as community resources (e.g., housing, employment, clothing, food, addiction and mental health treatment, affordable male and female condoms) for female ex-offenders.

## Authors’ original submitted files for images

Below are the links to the authors’ original submitted files for images. Authors’ original file for figure 1
Authors’ original file for figure 2

## Abbreviations

HIV: Human immunodeficiency virus
STI: Sexually transmitted infection.
