# Supplementary material for: Effects of incarceration on risky Sex: focus group data from Two New England states
Source: Health Justice. 2014 Apr 2;2:8. doi: 10.1186/2194-7899-2-8 (PMC5151510; doi:10.1186/2194-7899-2-8)
Supplement: Supplementary file 1 — Authors’ original file for figure 1 [file 40352_2013_8_MOESM1_ESM.pdf]

Table 1. Structure of Moderated Focus Groups

Topic	Estimated Time	Question Examples
Introduction	5 minutes	• Basic Introduction
HIV Risk	25 minutes	• How do women choose sexual partners when they leave prison?<br>• What's the goal for sex?<br>• Do women see the same men from before coming to prison?<br>• Do you think that they are good partners and what makes them so?<br>• Are they not so good? What makes them bad partners?<br>• Do you think women leaving prison use protection?<br>• Do you think women can talk to their men about whether other women are in the mix?<br>• Do you believe that woman have problems protecting themselves?<br>• What about male partners who are using drugs or drinking, is it hard for women to use protection then?<br>• Do you believe that women have problems protecting themselves when they use drugs?<br>• How do women get HIV?<br>• How can women protect themselves from HIV?
Condoms	20 minutes	• Why don't women use condoms more?<br>• Can you make (condoms) sexy?<br>• Do you think women are afraid of partners being violent when women bring up condom use?
Sexual Situations, Safe Sex and Violence	30 minutes	• When women want to use protection but don't, what gets in their way?<br>• When women are about to have sex, what emotions do they feel?<br>• Does have a supportive person in your life change how confident you feel asking a partner to use protection?<br>• What happens when women have had violence in their lives?<br>• Who controls sexual situations?<br>• What can women do to stay strong and empower themselves? How do women stay strong?
The End	10 minutes	• Summary
