# Supplementary material for: Effects of incarceration on risky Sex: focus group data from Two New England states
Source: Health Justice. 2014 Apr 2;2:8. doi: 10.1186/2194-7899-2-8 (PMC5151510; doi:10.1186/2194-7899-2-8)
Supplement: Supplementary file 2 — Authors’ original file for figure 2 [file 40352_2013_8_MOESM2_ESM.pdf]

Table 2. Themes and Sub-themes

Number	Theme Name
1	Changing Attitudes about Sex because of Incarceration
1a	Less interested in sex
1b	More interested in sex
2	Concerns about partner's faithfulness while one is incarcerated
3	Sex for survival and sex as a coping mechanism
4	Reported Mental Health Issues
5	General thoughts on condom use and safe sex
5a	Idea of wanting to become infected
5b	Being afraid and fearful of HIV and STIs
6	HIV/STI Testing
7	Strategies to getting a male partner to use a condom after incarceration
8	Reasons why women use condoms
9	Reasons why women do not use condoms
10	The relationship between drug use and sexual behavior, with condom use
11	Number of sex partners, before and after release
11a	Monogamous relationships
11b	Multiple sexual partners
12	Relationship with children
12a	Children has a motivator to stay clean and to improve life after prison
12b	Desire to teach children about safe sex
13	Communication with sexual partners about sex
14	Empowerment
15	Past Family Environment, Abuse, and Domestic Violence
16	Access to community resources post release
17	Interpersonal violence and abuse
18	Coping Skills
